# Amyotrophic lateral sclerosis and chronic inflammatory demyelinating polyneuropathy coexistence in a patient with a C9orf72 variant: case report

**DOI:** 10.3389/frdem.2026.1785124

**Published:** 2026-04-13

**Authors:** Christopher File, Anthony M. Price, Rowaid Ahmad, Elena Shanina, Ruiqing L. Sun

**Affiliations:** Department of Neurology, The University of Texas Medical Branch at Galveston, Galveston, TX, United States

**Keywords:** amyotrophic lateral sclerosis, chronic inflammatory demyelinating polyneuropathy, concurrent diagnosis, electromyography, immune-mediated neuropathy, neurodegenerative disorder, neuromuscular disorder, sensory-motor polyneuropathy

## Abstract

**Background:**

The *C9orf72* variation has been strongly implicated in the inheritance of familial ALS, frontotemporal dementia (FTD), and combined ALS-FTD cases. Increasing evidence implicates immune changes and inflammation in some ALS patients. Several studies demonstrated that ALS coexists with CIDP or polyneuropathy. Mouse models of C9orf72 loss-of-function mutations exhibit fatal immune dysregulation.

**Case summary:**

A 62-year-old Caucasian man developed right foot drop, and he underwent fibular nerve release without significant improvement. At the same time, he developed progressive weakness and numbness in his bilateral hands. MRI revealed cervical canal stenosis and neuroforaminal narrowing that prompted neurosurgical decompression without clinical improvement. Subsequently, he developed left foot drop. At the clinic presentation, he exhibited dysarthria, tongue fasciculations, weakness in all extremities, muscle atrophy, widespread fasciculations, and upper extremity hyperreflexia, meeting clinical criteria for ALS. Genetic testing identified a pathogenic variant in the C9orf72 gene, confirming a C9orf72 variant, commonly linked to familial ALS. Brain MRI demonstrated the motor band sign. Although EMG/NCS findings were consistent with lower motor neuron disease, he also had signs of demyelinating polyneuropathy based on conduction parameters. Neuromuscular ultrasound showed significant multifocal nerve enlargement typical of immune-mediated neuropathy. CSF studies revealed albuminocytologic dissociation (protein: 112 mg/dL, with normal cell count) and high albumin quotient and index. He fulfilled the 2021 EAN/PNS criteria for possible typical CIDP. He was treated with intravenous immunoglobulin in addition to riluzole with temporary improvement.

**Conclusion:**

This is the first case of the co-existence of CIDP and ALS in the setting of a pathogenic C9orf72 variant.

## 1 Introduction

Amyotrophic lateral sclerosis (ALS) is a progressive neurodegenerative disorder that primarily affects motor neurons in the brain and spinal cord. With an approximate incidence rate of 1–2.6 cases per 100,000 persons annually and an average age of 58–60, it is the most common motor neuron disease in adult populations (Talbott et al., 2016). Patients typically present with a combination of upper and lower motor neuron signs, including asymmetric limb weakness, spasticity, hyperreflexia, fasciculations, and difficulty with speech and swallowing (Zarei et al., 2015). There is a large, estimated delay in the diagnosis of ALS due to the non-specificity of the symptoms, the overlap with other neuromuscular diseases, and diagnosis uncertainty, especially when classic diagnostic symptoms of ALS are not present (Segura et al., 2023). Further difficulty in identifying ALS arises when other neurological disorders, such as multiple sclerosis, myasthenia gravis, dermatomyositis, polymyositis, and inflammatory polyneuropathies, concurrently occur in such patients (Longinetti et al., 2022).

Chronic inflammatory demyelinating polyneuropathy (CIDP), an inflammatory polyneuropathy, is characterized by chronic and multifocal sensorimotor deficits (Dimachkie and Barohn, 2013). The primary pathology of CIDP involves progressive demyelination of peripheral nerves, leading to symptoms such as muscle weakness, areflexia, and sensory disturbances (Dimachkie and Barohn, 2013). Diagnostic criteria for CIDP vary significantly, and multiple subtypes exist, but a commonality is peripheral motor demyelination, sensory demyelination, or both. Like ALS, clinical presentations vary significantly and can lead to diagnostic delays (Zarei et al., 2015). Albeit uncommon, CIDP has been observed to concurrently occur in patients with ALS (Longinetti et al., 2022).

Patients presenting with both conditions may exhibit signs of both upper and lower motor neuron degeneration alongside evidence of demyelination and axonal damage on electrodiagnostic studies. This overlap can obscure the diagnosis and delay treatment, as the immunomodulatory therapies effective for CIDP may not impact the progression of ALS (Zarei et al., 2015). The coexistence of ALS and CIDP is poorly characterized in the literature, and further research is needed to develop optimal management strategies for affected patients. Here, we present a patient diagnosed with ALS and overlapping CIDP following months of motor dysfunction initially attributed to orthopedic trauma. Interestingly, the patient met the criteria for both ALS and possible typical CIDP, supported by combined upper and lower motor neuron signs, electrodiagnostic study, neuromuscular ultrasound, and cerebrospinal fluid (CSF) albuminocytologic dissociation and C9orf72 hexanucleotide repeat expansion.

## 2 Case description

### 2.1 Patient presentation and past medical history

The patient is a 62-year-old Caucasian man who presented to our clinic for dysarthria, difficulty swallowing, weakness in both hands, and bilateral foot drop. He has had muscle fasciculation for 10–15 years.

The patient has a complicated medical history over many years involving musculoskeletal trauma and neurological issues. He has a history of left anterior cruciate ligament and left medial meniscus tears and repairs back 5 years ago. He also experienced a traumatic injury to his right leg approximately 3 years ago, in which his leg was trapped between rocks, causing multiple tears in unspecified right upper leg muscles. This trauma later caused a heterotopic ossification of the right hip, which orthopedic surgery subsequently removed and improved his hip function. However, the patient noticed his right foot dropping after his right hip surgery. Family history is significant because his mother has dementia of an unknown cause.

MRI of the cervical spine demonstrated degenerative changes with moderate spinal canal stenosis and bilateral foraminal narrowing. The patient underwent a fibular nerve release 9 months after his right foot drop and 1 year before clinical presentation, without appreciable improvement. At the same time, he noticed bilateral hand weakness and intermittent bilateral arm numbness, which was ascribed to cervical degenerative disease. The patient underwent an anterior cervical discectomy and fusion (ACDF) 1 month after his fibular nerve release. He noticed some temporary improvement in his hand strength, but later the symptoms got worse.

Over the following year, he developed worsening bilateral hand weakness, left foot drop, dysarthria, and recently intermittent difficulty swallowing episodes. He ultimately presented to our neurology clinic for evaluation at 4 months following the onset of the left foot drop. He endorsed urinary urgency and incontinence for the preceding 3 months. A few days after the initial encounter, he underwent a planned admission for open reduction and internal fixation of a right tibial fracture, after which there was no improvement in the foot drop. At his subsequent neuromuscular appointment approximately 2 months later, the patient complained of numbness/tingling in his hands and feet.

### 2.2 Investigations and diagnostics

His MoCA was 22/30 (he lost 5 points in visuospatial, 1 point in language, and 2 points in delayed recall). A neurological exam revealed mild lingual, buccal, and guttural dysarthria; bilateral distal muscle atrophy; split hand sign; widespread fasciculations including trunk muscles, bilateral UE, and LE weakness (more severe distally, 1/5–2/5 on the MRC scale); positive jaw jerk reflex; diffuse hyperreflexia in bilateral upper extremities and patella; positive cross-adduction and diminished ankle reflex bilaterally; and decreased sensation of pinprick, temperature, and vibration in bilateral extremities.

**Figure 1:**
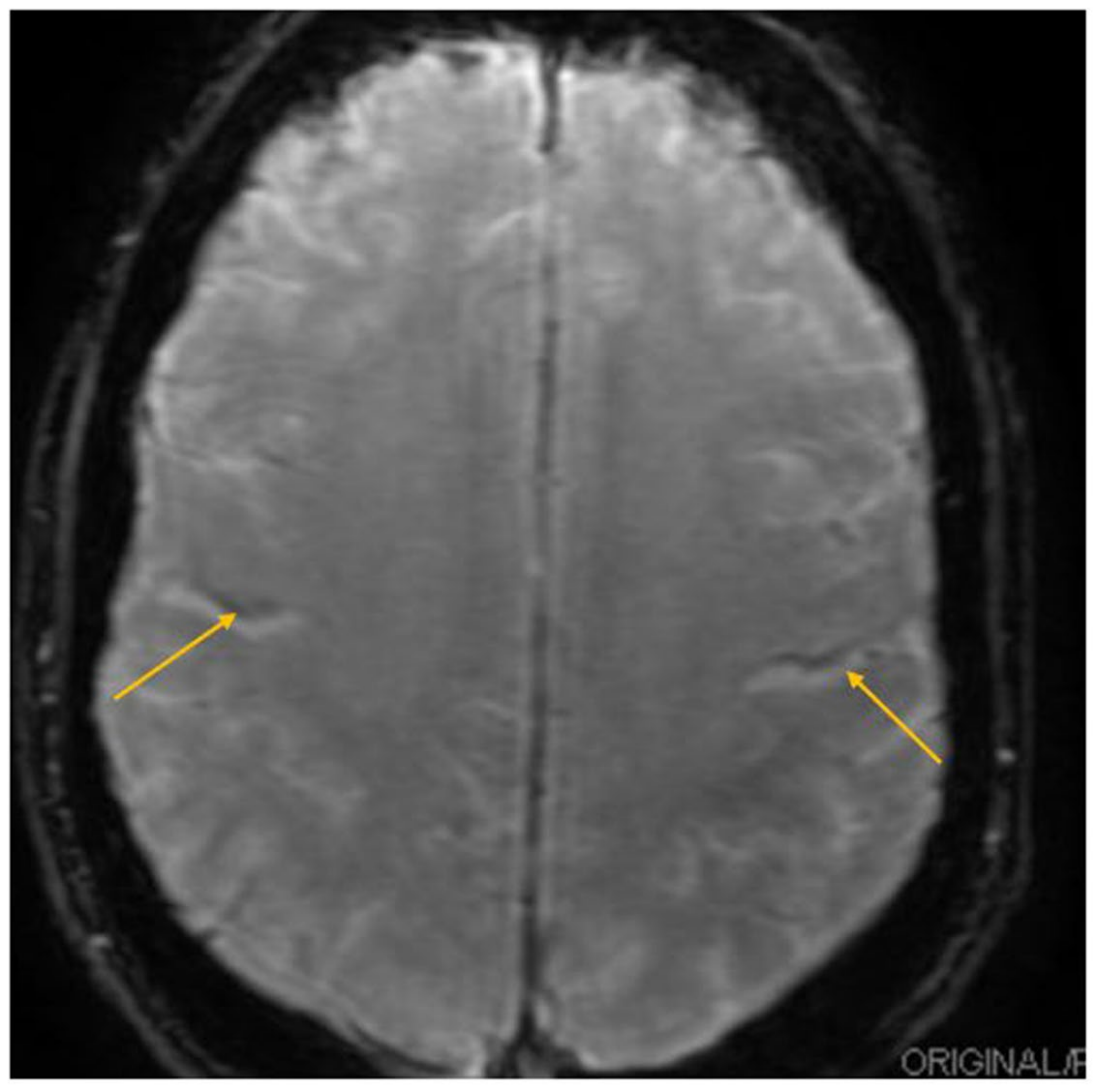
Brain MRI. MRI SWAN sequence showed motor band sign (arrows).

Brain MRI at SWAN sequence revealed hypointensity in the precentral gyrus bilaterally, known as the motor band sign (Figure 1).

**Figure 2:**
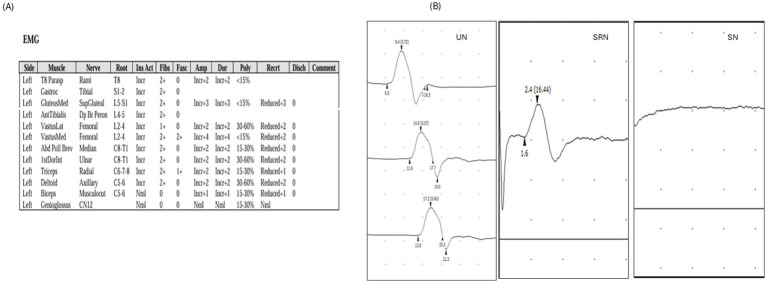
**(A)** Active denervation changes superimposed on very chronic denervation changes were noted in multiple myotomes of the upper and lower limb and thoracic paraspinal muscle. Genioglossus muscle showed no fibrillations. Evidence of severe sensory-motor polyneuropathy with demyelinating and axonal features. **(B)** Waveforms of the right ulnar motor conduction study (slowing of conduction), right superficial radial (reduced amplitude) and right sural sensory (absent response conduction study). UN, ulnar nerve; SRN, superficial radial nerve; SN, sural nerve.

**Figure 3:**
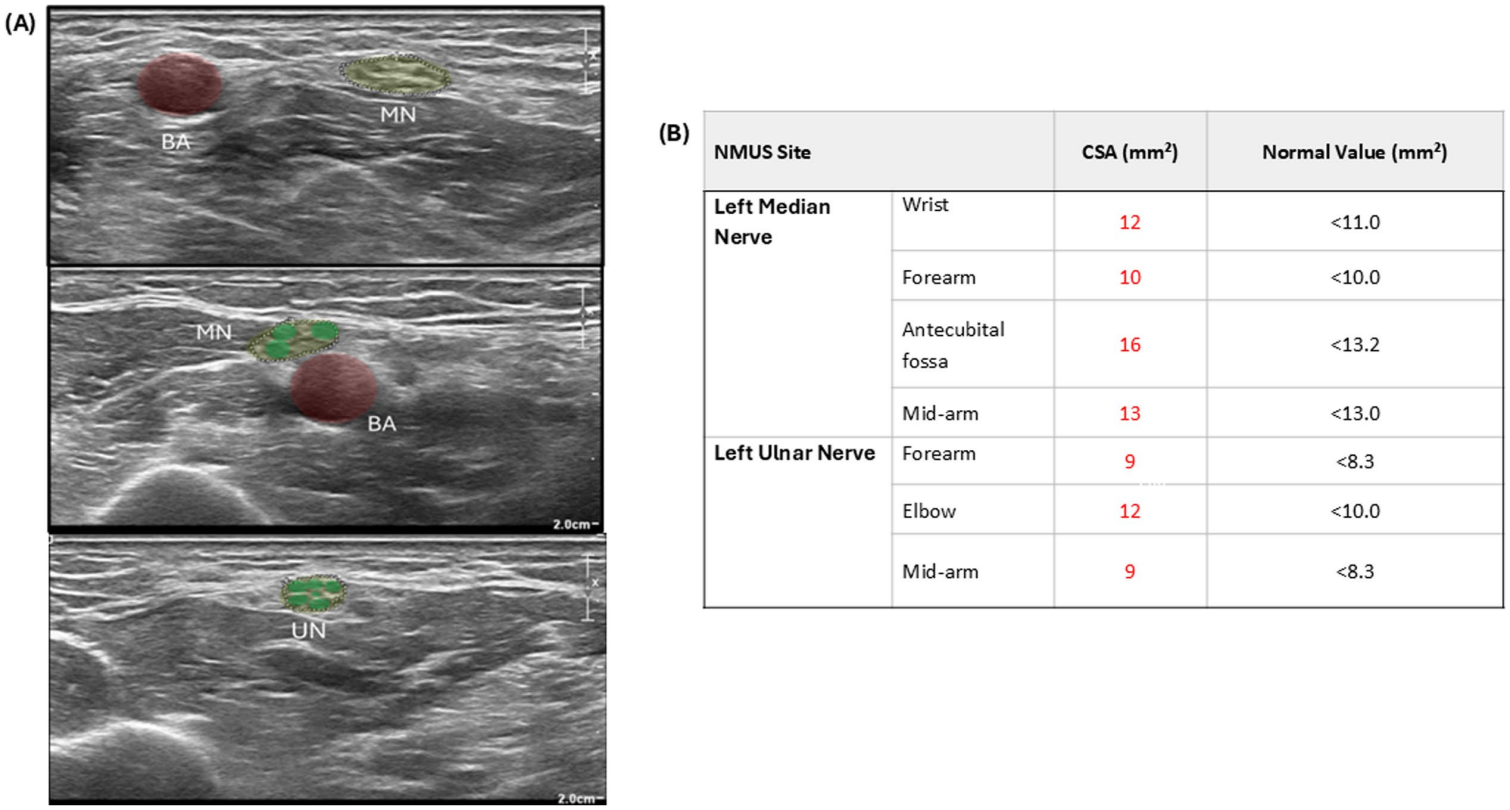
Sensory nerve conduction study (NCS) and neuromuscular ultrasound (NMUS) findings. **(A)** Sonographic images of the median and ulnar nerves (yellow) demonstrating enlargements outside compression sites and prominent fascicules (green). **(B)** Neuromuscular ultrasound measurements of cross-sectional areas of the median and ulnar nerves. MN-median nerve, UN-ulnar nerve, BA-brachial artery, CSA-cross-sectional area.

Due to clinical suspicion of ALS, the patient underwent nerve conduction studies (NCSs) and needle electromyography (EMG). A nerve conduction study showed absent fibular and tibial motor responses bilaterally, as well as absent right median motor responses (Table 1A). There were extensive active and chronic denervation changes in multiple myotomes in three regions (thoracic, cervical, and lumbosacral) (Figure 2A) consistent with the clinical impression of ALS. However, there were also significant abnormalities in sensory nerves: sural and superficial fibular responses were absent; radial, median, and ulnar sensory responses were reduced, consistent with superimposed polyneuropathy (Table 1B; Figure 2B). Furthermore, there was reduced conduction velocity and increased compound motor action potential (CMAP) duration in the right ulnar nerve, prolonged distal latency, and decreased CMAP amplitude in the left median and ulnar nerves (Table 1A; Figure 2B). The EMG/NCS results raised suspicion for chronic inflammatory demyelinating polyneuropathy (CIDP), prompting additional investigations, including neuromuscular ultrasound and CSF analysis as supportive criteria. Neuromuscular ultrasound showed multifocal enlargements of the median and ulnar nerves outside these compression sites (Figures 3A,B), which are often seen in immune-mediated neuropathies but not in ALS. CSF analysis showed elevated total protein at 112 mg/dL, with a normal cell count, an elevated albumin index, and an otherwise normal study. At follow-up, he developed decreased pinprick sensation, which progressed to diminished temperature and vibration sensation up to his knees bilaterally. Per the 2021 EAN/PNS criteria, possibly typical CIDP applies when a patient has a typical CIDP clinical phenotype but electrodiagnostic testing shows demyelinating motor conduction criteria in only one motor nerve (rather than ≥2 for “CIDP”), with sensory conduction abnormalities in at least two nerves. In our patient, NCS/EMG showed motor nerve demyelinating changes with sensory NCS abnormalities in multiple nerves, supported by CSF albuminocytologic dissociation (protein 112 mg/dL, normal cell count) and multifocal median/ulnar nerve enlargement on neuromuscular ultrasound, consistent with possible typical CIDP.

**Table 1:** Motor and sensory nerve conduction study (NCS).

(A)				
Nerve/stimulation site	Latency (NL) (ms)	Amplitude (NL) (mV)	Duration (NL) (ms)	Velocity (NL) (m/s)
Left median motor (APB)				
Wrist	4.5 (<4.0)	3.9 (>6.0)	5.23 (<7.8)	
Elbow	9.8	3.3	5	49 (>48)
Right median motor (APB)				
Wrist/elbow	No response	No response	No response	No response
Left ulnar motor (ADM)				
Wrist	5.4(<3.6)	3.1 (>6)	6.25 (< 8.5)	
Below elbow	8.4	5.4	7.27	52 (>51)
Above elbow	10.2	5.2	7.03	
Right ulnar motor (ADM)				
Wrist	4.9 (<3.6)	0.6 (>6)	10.7 (< 8.5)	36 (>51)
Below elbow	12.1	0.6	10.31	42 (>51)
Above elbow	14.5	0.6	8.67	
Right fibular motor (EDB and TA)	No response	No response	No response	No response
Right tibial motor (AHB)	No response	No response	No response	No response

(B)		
Sensory NCS stimulation site	Latency (ms)	Amplitude (μV)
Left median sensory	4.1 (<3.6)	21.5 (>15)
Right median sensory	4.7 (<3.6)	10.4 (>15)
Left ulnar sensory	3.5 (<3.1)	7.1 (>10)
Right ulnar sensory	4.0 (<3.1)	4.6 (>10)
Right superficial radial sensory	2.4(<2.9)	16.4 (>20)
Right superficial peroneal sensory	No response	No response
Right sural sensory	No response	No response

(A) NCS showed conduction slowing in the right ulnar nerve (wrist to elbow), prolonged distal latencies and increased CMAP duration in left median motor, bilateral ulnar motor, absent tibial, fibular, and right median motor responses. (B) NCS showed reduced median, ulnar, and superficial radial sensory responses and absent superficial perineal and sural sensory response. APB, abductor pollicis brevis muscle; ADM, abductor digiti minimi muscle; AHB, abductor hallicis brevis muscle; EDB, extensor digitorum brevis muscle; TA, tibialis anterior muscle.

His genetic test for ALS showed a positive real-time PCR for a C9orf72 variant, with >30.8 GGGGCC repeats (which is considered diagnostic for the variant). The patient was diagnosed with certainty with ALS and met the diagnostic criteria for possible typical CIDP, but it is more likely that inflammatory neuropathy is a manifestation of the disease spectrum than a simple coincidence. The patient was started on riluzole and immunomodulation with IVIG 0.5 g/kg/day infusions for 2 days per month for 6 months, with subjective transient improvement in symptoms, but continued to endorse LE weakness and progressive dysphagia, as substantiated by follow-up evaluation, with no changes in motor or sensory exam findings.

## 3 Discussion

### 3.1 Difficulties in identification and diagnosis

Although ALS is classically a motor neuron disorder, sensory involvement is increasingly recognized and can meaningfully obscure early clinical attribution (Rubio et al., 2022). The coexistence of ALS with a CIDP-like inflammatory demyelinating polyneuropathy has been described but remains rare, largely limited to isolated case reports/series and registry-level observations of ALS with concurrent neuroinflammatory disease (Longinetti et al., 2022; Sennfält et al., 2023; Akaishi et al., 2014; Rajabally and Jacob, 2008; Megens and de Souza, 2024; Echaniz-Laguna et al., 2006). Our case fits within this uncommon spectrum, with the development of definite ALS alongside electrophysiologic and supportive findings consistent with inflammatory demyelinating neuropathy.

Akaishi et al.’s patient, a 37-year-old woman, presented for years with a CIDP-forward phenotype: distal leg weakness/foot drop plus sensory symptoms, demyelinating features on NCSs approximately 5 years from the initial onset of gait imbalance; this was confirmed at that point by sural nerve biopsy changes (Akaishi et al., 2014). She had partial, objective responses to IVIG early, with diminishing benefits over time, and only later developed unequivocal ALS features culminating in death after a long (≈12 years) course. Though this patient had a confirmatory biopsy and NCSs to determine CIDP, clinically both Akaishi et al.’s patient and our case show the same overarching pattern seen in the overlapping literature: immunotherapy with only transient/partial benefit for progressive motor neuron disease.

Akaishi et al. continue to summarize 14 reported cases of ALS with concurrent CIDP-spectrum supportive findings with at most partial/transient immunotherapy benefit, followed by a clinical course ultimately dominated by progressive ALS, including quadriparesis and bulbar symptoms (Akaishi et al., 2014). In a study by Rajabally and Jacob, four patients presented with clinical features and investigation suggestive of neuropathy and eventually progressed to the ALS-predominated clinical presentation; none of them had a significant objective response to immunomodulatory treatment (Rajabally and Jacob, 2008). Although sensory involvement has been reported in ALS, Akaishi et al. and Rajabally and Jacob emphasize that disproportionate sensory symptoms/signs can characterize ALS–CIDP overlap presentations. In our patient, clinical sensory complaints with multi-nerve sensory NCS abnormalities, motor nerve EMG/NCS abnormalities, and supportive ancillary features supported a CIDP-spectrum process superimposed on ALS (Rubio et al., 2022; Akaishi et al., 2014; Rajabally and Jacob, 2008).

In contrast, Megens and de Souza recently described a patient with clinically typical ALS (UMN + LMN signs), but CIDP-like demyelinating findings on NCSs, where the motor band sign supported ALS, and immunotherapy was ineffective (Megens and de Souza, 2024). They note that the coexistence of ALS and a demyelinating neuropathy is rare and that the relationship between the two is not well established. In comparison, our case showed stronger evidence of a CIDP-spectrum process, supported by sensory symptoms, progressive objective sensory loss, multi-nerve sensory NCS abnormalities, albuminocytologic dissociation, and multifocal median and ulnar nerve enlargement on neuromuscular ultrasound. These features are far more characteristic of immune-mediated neuropathy than ALS alone.

As several colleagues have previously noted, distinguishing clinical findings of ALS from those of other conditions can be challenging, especially when hallmark signs are obscured by a complex clinical picture. ALS is inherently heterogeneous, and there are no disease-specific biomarkers. In our case, the patient had an extensive history of orthopedic trauma overlapping with other neurological issues. He was initially diagnosed with fibular neuropathy and cervical stenosis and subsequently underwent right fibular nerve release and ACDF, but neither procedure improved his symptoms. In retrospect, it is clear that these surgeries were performed for symptoms ultimately attributable to ALS. Early diagnosis of limb-onset ALS is very challenging because initial symptoms commonly mimic mononeuropathy, cervical radiculopathy, or myelopathy. Sennfält et al. reported that limb-onset ALS is associated with the greatest diagnostic delay, often due to misdiagnosis as degenerative spine disease or peripheral neuropathy, with an average time from onset to diagnosis of 12.9–16 months (Sennfält et al., 2023). Our patient was diagnosed 25 months after his initial right foot drops and 14–15 months after his fibular nerve release and cervical ACDF.

One year later, after our patient’s surgery, his brain MRI demonstrated the motor band sign, which is a radiological sign described in ALS. His genetic testing confirmed a C9orf72 variant, commonly linked to familial ALS. At the same time, he developed typical ALS signs and symptoms; furthermore, an electrophysiological study supports ALS and possible CIDP. It is argued that the patient might have diabetic neuropathy, M-protein neuropathy, or autoimmune nodopathy. The fact is that the patient did not have diabetes and had negative M-protein screening, which ruled out diabetic neuropathy and monoclonal protein neuropathy. However, we did not have results available for anti-NF155 or anti-CNTN1, which would rule out autoimmune nodopathy.

### 3.2 Possible combined mechanisms

The occurrence of both ALS and CIDP in the same patient is documented but considered very rare (Zarei et al., 2015; Longinetti et al., 2022; Akaishi et al., 2014; Rajabally and Jacob, 2008; Megens and de Souza, 2024; Echaniz-Laguna et al., 2006; Isaacs et al., 2007). Several case reports have shown concurrent sensory neuropathies or other neuroinflammatory disorders with ALS. A retrospective chart review of patients with confirmed ALS diagnosis indicated that, of 151 ALS patients, 28 had a concurrent neuroinflammatory disorder, including multiple sclerosis, myasthenia gravis, inflammatory polyneuropathy, or dermatopolymyositis. Of these 28, only 4 had inflammatory polyneuropathy (Longinetti et al., 2022).

Patients presenting with concurrent ALS and CIDP-like features vary widely, with many dying within 3–5 years of symptom onset, and fewer surviving beyond 6 years at follow-up (Akaishi et al., 2014; Rajabally and Jacob, 2008; Megens and de Souza, 2024; Echaniz-Laguna et al., 2006; Isaacs et al., 2007). A consistent feature among these cases is the lack of response to corticosteroids (Akaishi et al., 2014; Isaacs et al., 2007). Occasional, short-lived improvements have been reported with IVIG, but in most cases, the benefit is transient or absent (Akaishi et al., 2014; Echaniz-Laguna et al., 2006).

Pathological studies in patients with coexisting ALS and CIDP have revealed significant and overlapping features of both conditions, as demonstrated in the work of both Echaniz-Laguna et al. and Rajabally et al. In Echaniz-Laguna’s study, patients exhibited severe neuronal loss in the anterior horn cells, axonal loss in the corticospinal tracts, and endoneurial infiltration of mononuclear cells, primarily T-lymphocytes and macrophages, in the lumbar roots and peripheral nerves (Echaniz-Laguna et al., 2006). This infiltration was associated with demyelination and axonal loss, consistent with CIDP, while the spinal cord pathology clearly indicated ALS. Similarly, Rajabally et al. reported patients initially diagnosed with CIDP-like disorders who later progressed to a definitive ALS diagnosis (Rajabally and Jacob, 2008). These patients showed segmental demyelination on teased-fiber studies, with one case demonstrating elevated CSF protein levels typical of CIDP. Despite immunomodulatory treatments, all patients experienced progressive deterioration, evidenced by the severe neuronal and axonal degeneration observed postmortem (Akaishi et al., 2014; Rajabally and Jacob, 2008; Megens and de Souza, 2024; Echaniz-Laguna et al., 2006).

The *C9orf72* repeat expansion has been heavily implicated in the inheritance of familial ALS, frontotemporal dementia (FTD), and combined ALS-FTD cases (De Marchi et al., 2023; Sakkas et al., 2017). While the function of its protein product has yet to be determined, it has been linked to immune dysregulation and autoimmunity. Specifically, the lack of function disrupts autophagy and lysosomal pathways, leading to the accumulation of inflammatory aggregates and promoting inflammatory pathways such as NF-κB and the NLRP3 inflammasome. The presence of heightened inflammatory responses in ALS patients with *C9orf72* expansion variants has been associated with elevated levels of pro-inflammatory cytokines and the production of autoantibodies (De Marchi et al., 2023; Sakkas et al., 2017). In our patient, this inflammatory milieu may contribute to the development of CIDP.

Whether demyelinating polyneuropathy was coincident with ALS or was a cause or consequence of motor neuron degeneration in this patient remains to be elucidated. However, we believe that ALS might be a multisystem disorder, which includes neurodegenerative and immune-mediated mechanisms, as colleagues have alluded to Rajabally and Jacob (2008), Echaniz-Laguna et al. (2006), Isaacs et al. (2007), and Dziadkowiak et al. (2024). The exact pathophysiological relationship between these two conditions remains poorly understood; further studies are warranted to better understand the intersection between neurodegenerative and autoimmune mechanisms in such cases.

## 4 Conclusion

This case demonstrated a delayed ALS diagnosis due to a variable ALS clinical presentation, which underscores the necessity for heightened clinical awareness and thorough diagnostic workups in patients presenting with complex neuromuscular syndromes. This is the first case of the co-existence of CIDP and ALS in the setting of C9orf72 variation. Further research is needed to determine whether there is a causal link between ALS and CIDP, especially in the setting of the C9orf72 variant, potentially unveiling novel therapeutic targets and improving patient outcomes.

## Data Availability

The raw data supporting the conclusions of this article will be made available by the authors, without undue reservation.
